# Associations of ventriculomegaly and white matter hyperintensities with glymphatic dysfunction in idiopathic normal pressure hydrocephalus

**DOI:** 10.1007/s00330-024-11320-3

**Published:** 2025-01-21

**Authors:** Qian Wu, Jiakuan Chen, Xiaolin Yang, Xiejun Zhang, Wenjie He, Jun Xia

**Affiliations:** 1 Department of Radiology, The First Affiliated Hospital of Shenzhen University, Shenzhen University, Shenzhen Second People’s Hospital, Shenzhen, China; 2 Department of Radiology, South China Hospital, Medical School, Shenzhen University, Shenzhen, China; 3 Longgang Central Hospital of Shenzhen, Shenzhen, China; 4 Department of Neurosurgery, The First Affiliated Hospital of Shenzhen University, Shenzhen University, Shenzhen Second People’s Hospital, Shenzhen, China

**Keywords:** Glymphatic system, Diffusion tensor imaging, Hydrocephalus

## Abstract

**Objectives:**

To investigate glymphatic function in idiopathic normal pressure hydrocephalus (iNPH) using the diffusion tensor image analysis along the perivascular space (DTI-ALPS) method and to explore the associations of ALPS index with ventriculomegaly and white matter hyperintensities (WMH).

**Materials and methods:**

This study included 41 patients with iNPH and 40 age- and sex-matched normal controls (NCs). All participants underwent brain MRI. Based on DTI, we then calculated the ALPS index to obtain the water diffusivity along the perivascular space. Ventricular volume and WMH were also determined. Differences in the diffusivities and ALPS indexes between the iNPH and NC groups were investigated; associations of the DTI-ALPS index with ventriculomegaly and WMH were analysed.

**Results:**

Patients with iNPH had a lower ALPS index than NCs (*p* < 0.001). The ALPS index was significantly correlated with the normalised ventricular volume (*r* = −0.446, *p* = 0.004), but not with total WMH volume (*r* = −0.246, *p* = 0.126). Further regression analyses indicated that the reduced ALPS index was associated with increased ventricular volume (β = −7.158, *p* = 0.016), but not with normalised WMH volume (β = −2.796, *p* = 0.161). The receiver operating characteristic analysis demonstrated the ALPS index’s excellent diagnostic performance for iNPH (the optimal cut-off point = 1.322; sensitivity, 100.0%; specificity, 87.5%; AUC = 0.980).

**Conclusions:**

Patients with iNPH had a lower ALPS index, which may suggest impaired glymphatic function. This study demonstrated an association of DTI-ALPS index with ventriculomegaly, but not WMH in patients with iNPH.

**Key Points:**

***Question*** *Glymphatic dysfunction is crucial in idiopathic normal pressure hydrocephalus (iNPH) development, yet its associations with neuroimaging features remains unclear.*

***Findings*** *Diffusion tensor image analysis along the perivascular space (DTI-ALPS) revealed a reduced ALPS index in idiopathic normal pressure hydrocephalus, negatively correlating with ventricular volume.*

***Clinical relevance*** *DTI-ALPS enables non-invasive assessment of glymphatic function and its relationship with neuroimaging characteristics in idiopathic normal pressure hydrocephalus, facilitating the investigation of glymphatic dysfunction in iNPH pathophysiology.*

## Introduction

Idiopathic normal pressure hydrocephalus (iNPH) is characterised by non-obstructive ventriculomegaly and gait disorder, cognitive deficits, and urinary dysfunction [1]; however, its aetiology remains unclear. Notably, ventricular dilatation and white matter hyperintensity (WMH), the most common imaging signs of iNPH, are associated with drainage disorders of the cerebrospinal and interstitial fluids [2].

The glymphatic system, which depends on convective fluid transport through the perivascular and interstitial spaces and plays a role in clearing brain metabolites, is implicated in various diseases, including Alzheimer’s disease, Parkinson’s disease, diabetes, and iNPH [3]. Several studies have revealed that decreased glymphatic function is associated with iNPH [4, 5], and early applications of glymphatic magnetic resonance imaging (MRI) have demonstrated impaired glymphatic clearance and transependymal migration of contrast agents in iNPH [4]. However, this method is invasive, and multiple MRI acquisitions are required before and after intrathecal administration of the contrast agents, a process that can be challenging for patients to tolerate. Recently, Taoka et al [5] proposed a non-invasive method called “diffusion tensor image analysis along the perivascular space” (DTI-ALPS) to assess glymphatic function in the human brain. The ALPS index proposed in that study reflects the water diffusion along the perivascular space and has been used to assess the activity of the glymphatic system. The study indicated that the ALPS index was positively correlated with the severity of cognitive dysfunction in Alzheimer’s disease. Subsequently, the ALPS index has been applied in patients with dementia [6], Parkinson’s Disease [7], and type 2 diabetes mellitus [8]. However, to our knowledge, only three studies with small sample sizes have explored glymphatic system activity in patients with iNPH using the DTI-ALPS method [9–12]. These studies demonstrated that patients with iNPH had a lower ALPS index than normal controls (NCs), and this reduced ALPS index was associated with ventricular enlargement [9, 12]. WMH is prevalent in patients with iNPH, presenting as interstitial oedema, and may be associated with glymphatic dysfunction. However, whether the ALPS index is independently associated with ventricular enlargement and WMH remains unclear.

Therefore, this study aimed to assess alterations in the glymphatic function in patients with iNPH based on DTI and investigate the association of the glymphatic function with WMH and ventriculomegaly.

## Materials and methods

### Participants

**Fig. 1:**
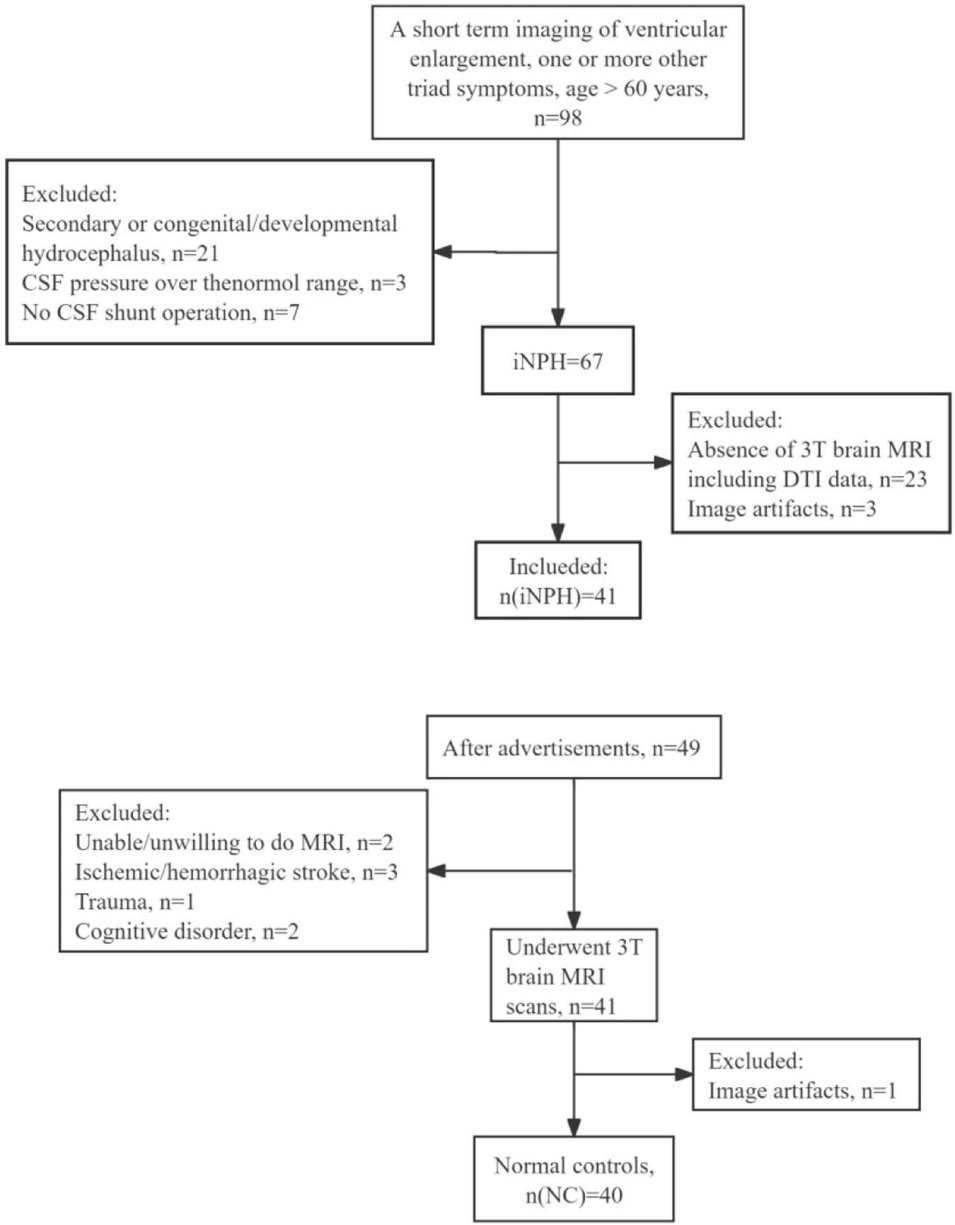
Flowchart of selection for the iNPH and NC groups in this study. iNPH, idiopathic normal pressure hydrocephalus; NC, normal control

Written informed consent was obtained from all participants before examinations. The study protocol was approved by the Hospital Bioethics Committee. Notably, 41 patients with iNPH who were referred to the Department of Neurology at our institution between January 2019 and March 2022, as well as 40 healthy controls, were recruited to the study. All patients fulfilled the criteria of the updated Japanese guidelines for iNPH [13, 14] and were eligible for cerebral spinal fluid (CSF) shunt operation. Figure 1 shows the flowchart of the selection process for the iNPH and NC groups. A neurologist with 15 years of experience in movement disorders performed the clinical assessment according to the guidelines for iNPH. Initially, 98 patients were included in this study based on the following criteria: age > 60 years, presenting with one or more typical triad symptoms (gait disturbance, cognitive disorder, and urinary incontinence), and presence of recent radiological evidence of ventricular enlargement. However, 57 participants were excluded due to secondary or congenital/developmental hydrocephalus (*n* = 21), absence of CSF shunt operation (*n* = 7) or CSF pressure exceeding the normal threshold (*n* = 3), absence of 3-T brain MRI including DTI (*n* = 23), or image artefacts (*n* = 3).

During the same period, an age- and sex-matched NC group was recruited by advertising in several health management centres, targeting older individuals (age > 60 years) without a definite history of significant medical conditions, such as ischemic or haemorrhagic stroke, trauma, or cognitive disorder. Therefore, 40 age- and sex-matched healthy volunteers were selected for the NC group following a thorough medical history review. Notably, all recruited participants were right-handed and underwent clinical assessments.

### MRI protocol

All MRI data were obtained using a 3-T MRI scanner (Siemens Prisma). The participants’ heads were immobilised with foam pillows inside the coil to reduce motion artefacts. DTI sets with b = 1000 (echo planar, repetition time (TR) = 9500 ms, echo time (TE) = 90 ms, motion probing gradients = 64 directions, field of view (FOV) = 240 × 240 mm^2^, matrix = 128 × 128 mm^2^, slice thickness = 3 mm) were acquired alongside conventional morphological images. The following sequences were obtained using routine protocols: sagittal three-dimensional (3D) T1-weighted imaging (T1-WI) magnetisation-prepared rapid acquisition gradient-echo (MPRAGE) sequence, sagittal 3D fluid-attenuated inversion recovery (FLAIR) imaging with axial reconstruction, axial T2-WI, and susceptibility weighted imaging (SWI). Imaging parameters were as follows: (1) sagittal 3D T1-WI: TR = 2300 ms; TE = 3.55 ms; slice thickness, 0.9 mm; flip angle, 8°; FOV = 240 × 240 mm^2^; acquisition matrix = 256 × 256 mm^2^; (2) 3D FLAIR: TR = 4800 ms; TE = 274 ms; slice thickness, 1.5 mm; flip angle, 90°; FOV = 240 × 240 mm^2^; acquisition matrix = 240 × 240 mm^2^; (3) axial T2-WI: TR = 3000 ms; TE = 80 ms; slice thickness, 5 mm; flip angle, 90°; FOV = 180 × 230 mm^2^; acquisition matrix = 420 × 375 mm^2^; (4) SWI: multi-echo fast-field-echo sequence; TR = 41 ms; total 4 echoes; first TE = 7.2 ms; echo interval, 6.2 ms; slice thickness, 2 mm; flip angle, 20°; FOV = 200 × 220 mm^2^; acquisition matrix = 384 × 384 mm^2^.

### DTI-ALPS processing and image analysis

Diffusion metric images from DTI were processed using FMRIB Software Library (FSL) version 5.0.9 (https://fsl.fmrib.ox.ac.uk/). We computed diffusivity maps in the directions of the *x*-axis (right-left, Dx), *y*-axis (anterior-posterior, Dy), and *z*-axis (inferior-superior, Dz). Fractional anisotropy (FA) maps were created and aligned into the FMRIB58_FA standard space using FSL’s linear and nonlinear registration tools.

**Fig. 2:**
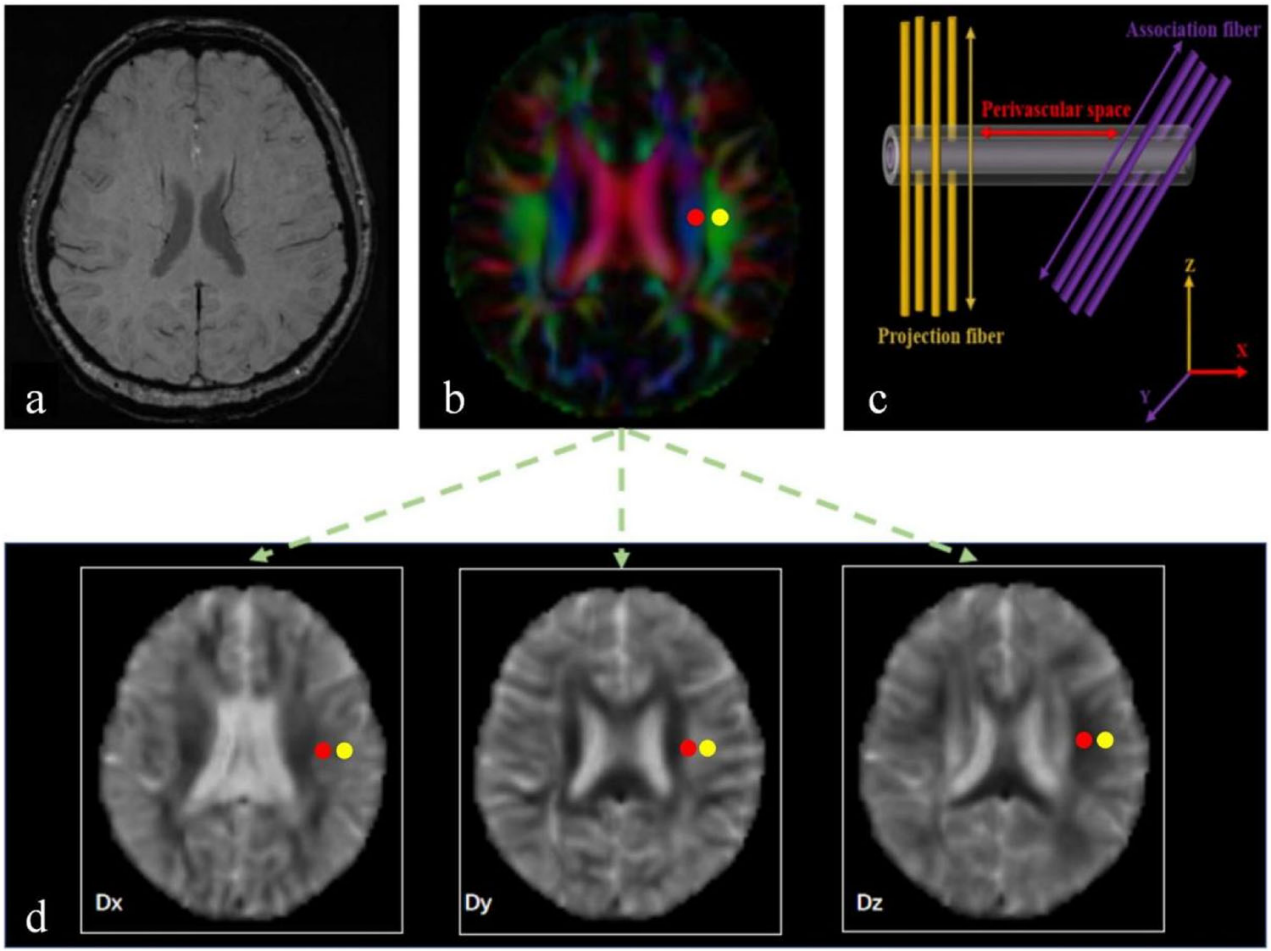
Schematic drawing of the diffusivity measurement using the DTI-ALPS methods. **a** Axial SWI on the slice at the level of the lateral ventricle body indicating that parenchymal vessels run laterally (*x*-axis). **b** Colour-coded FA map of DTI showing the distributions of the projection neural fibres running along the *z*-axis (blue colour) and the association neural fibres along the *y*-axis (green colour) areas. Two ROIs were placed in the areas with the projection (red circle) and association areas (yellow circle) to measure diffusivities of the three directions (*x*, *y*, *z*) on a colour-coded FA map. **c** Schematic diagram indicating the relationship between the direction of the perivascular space (grey cylinders) and the directions of the fibres. Note that the direction of the perivascular space is perpendicular to both projection and association fibres. **d** Two ROIs on colour-coded FA maps were then copied and pasted onto the three diffusivity maps to measure diffusivities along the *x*-, *y*-, and *z*-axes. DTI-ALPS, diffusion tensor image analysis along the perivascular space; SWI, susceptibility-weighted imaging; ROIs, regions of interest; FA, fractional anisotropy

Initially, referencing SWI, an axial slice was selected at the level of the lateral ventricular body, where the trans-medullary vessels passed perpendicularly to the ventricle. At this level, the direction of the perivascular space was perpendicular to the ventricular wall (mostly along the *x*-axis). This direction was also perpendicular to the direction of the projection (mostly along the *z*-axis) and association fibres (mostly along the *y*-axis) (Fig. 2). Therefore, the diffusivity along the *x*-axis in the regions with projection/association fibres at least partly represents the diffusivity along the perivascular space. The areas of these neural fibres were identified on a colour-coded FA map using different FA values. Similar to Taoka et al all measurements were performed in the left hemisphere since all participants were right-handed. However, in contrast to Taoka et al we were concerned about more crossed nerve fibres in the area of the subcortical fibres; therefore, we chose to place two 5-mm-diameter spherical regions of interest (ROIs) in the areas of projection and association neural fibres (Fig. 2). These ROIs were registered using the same FA template. Diffusivity in the direction of the *x*-axis (Dx), *y*-axis (Dy), and *z*-axis (Dz) within the ROIs was obtained for each participant.

Finally, we calculated the ALPS index using the algorithm by Taoka et al [5]: ALPS index = (mean [Dx_proj_] + mean [Dx_assoc_])/(mean [Dy_proj_] + mean [Dz_assoc_]). An ALPS index close to 1.0 reflects minimal diffusion along the perivascular space, whereas higher values indicate greater diffusivity. Two trained neuroradiologists blinded to the clinical information of participants independently assessed all diffusion metrics. Interobserver consistency of diffusivities and ALPS indexes was assessed, and all diffusivities and ALPS indexes were then averaged and used for further analysis when consistency was good.

### Ventricular volumetry and WMH assessment

Our research team chose MPRAGE data for manual segmentation for ventricular volumetry. The specific manual delineation of the ventricle volume (VV) and total intracranial volume (ICV) process is as follows: (1) a radiologist with > 5 years of clinical work experience manually marked the VV and ICV used the ITK software (v3.8.0-RC1; http://www.itksnap.org) to label the ventricles; (2) Furthermore, a neurosurgeon with > 10 years of clinical work experience reviewed and adjusted the manual annotation results. The total VV was divided by ICV to obtain normalised VV (VV/ICV). The WMH lesion volume was calculated for each patient from the generated lesion mask using FSLstats, and WMH lesion masks were created on FLAIR images using the lesion prediction algorithm [15] and the Statistical Parametric Mapping–Lesion segmentation toolbox subsequent to lesion identification by an experienced neuroradiologist. After lesion filling using the FSL lesion filling toolbox [16], normalised brain and grey matter volumes were assessed from T1-weighted images using Structural Image Evaluation and Normalization of Atrophy for cross-sectional data, which is part of the FSL [17]. The WMH lesion masks and lesion-filled T1-weighted images were visually checked for accuracy. Similarly, considering individualised differences in ICV, the total WMH volume was divided by ICV to obtain normalised WMH volume (WMH volume/ICV). Furthermore, periventricular white matter hyperintensity (pWMH) and deep white matter hyperintensity (dWMH) were evaluated by an experienced neuroradiologist using the Fazekas scale [18] based on axial FLAIR imaging. pWMH was graded as follows: grade 0, absence; grade 1, caps or pencil-thin lining; grade 2, smooth halo; and grade 3, extension into the deep white matter. dWMH was graded as follows: grade 0, absence; grade 1, punctate foci; grade 2, beginning confluence; and grade 3, large confluence.

### Statistical analysis

All statistical analyses were performed using SPSS software (version 25.0; IBM Corporation). The Shapiro–Wilk test was used to assess normality. Clinical and structural MRI characteristics of patients with iNPH and controls were compared using unpaired *t*-tests (for normally distributed continuous variables) or the Mann–Whitney U test (for non-normally distributed variables), and differences in frequencies were compared using Chi-square tests. The consistency of the diffusivities and ALPS indexes between two observers was tested with intraclass correlation coefficients.

Receiver operating characteristic (ROC) curve analysis was employed to compare the diagnostic performance of the ALPS index between control and iNPH groups. The correlation between the diffusivities, ALPS index, WMH volume, ventricular volume, and the degree of pWMH and dWMH was determined using Spearman’s correlation coefficient. We performed multivariate linear regression analyses on normalised ventricular and WMH volumes. For all regression analyses, critical confounding factors, such as age and sex, were adjusted. The number of covariates was limited to three due to the small sample size. Statistical significance was set at *p* < 0.05.

## Results

### Clinical and structural MRI characteristics of participants

Forty-one patients with iNPH and forty sex- and age-matched NCs were finally included in this study. Table 1 summarises the clinical and structural MRI characteristics of the control and iNPH groups. There were no significant differences in sex or age between the two groups (*p* > 0.05). However, there were significant differences in the total WMH volume, normalised WMH volume, and pWMH and dWMH scores (all *p* < 0.001).

**Table 1:** Clinical characteristics and structural MRI characteristics of the NC and iNPH groups

Characteristics	NC (*n* = 40)	iNPH (*n* = 41)	*p*-values
Year, mean (SD)	67.4 (4.6)	68.6 (5.9)	0.311
Sex, male, *n* (%)	17 (42.50%)	23 (56.10%)	0.855
Symptoms present, *n* (%)			
Triad of symptoms	/	18 (43.9%)	
Gait + cognitive impairment	/	13 (31.7%)	
Gait + urinary symptom	/	7 (17.1%)	
Cognitive impairment + urinary symptom	/	3 (7.3%)	
Structural MRI characteristics			
WMHv, mL, median [range]	1.03 [0.84–2.17]	36.80 [4.91–81.93]	< 0.001*
WMHv/ICV, %, median [range]	0.10 [0.09–0.17]	2.59 [0.33–5.80]	< 0.001*
pWMH grade, *n* (%)			
Grade 3	0	13 (31.7%)	< 0.001*
Grade 2	0	21 (51.2%)	
Grade 1	32 (80.0%)	5 (12.2%)	
Grade 0	8 (20.0%)	2 (4.9%)	
dWMH grade, *n* (%)			
Grade 3	0	14	< 0.001*
Grade 2	6	21	
Grade 1	16	5	
Grade 0	18	1	
Total VV, mL, mean (SD)	/	125.0 (38.6)	
VV/ICV, %, median [range]	/	8.6 [5.6–13.6]	

Continuous variables with normal distribution are expressed as mean (standard deviation), continuous variables with skewed distribution are expressed as median [range], and categorical variables are expressed as number of cases (percentage)

*NC* normal control, *iNPH* idiopathic normal pressure hydrocephalus, *WMHv* WMH volume, *pWMH* periventricular white matter hyperintensities, *dWMH* deep white matter hyperintensities, *VV* ventricular volume, *ICV* intracranial volume, / not applicable

* *p*-values less than 0.05 indicate statistical significance

### Comparison of diffusivities and ALPS indexes between groups

The interobserver agreements on the diffusivities and ALPS indexes for both NC and iNPH groups between the two readers were good, with all interclass correlation coefficients > 0.7 (Table 2). Dx_proj_ and Dz_proj_ were significantly higher in the left projection fibre region in the iNPH group than in the NC group (*p* < 0.05); however, the ALPS index was significantly reduced in the iNPH group than in the control group (*p* < 0.001) (Table 3).

**Table 2:** Interobserver agreement on the diffusivities and ALPS index in the two groups

		Dx_proj_ [× 10^−3^mm^2^/s]	Dx_assoc_ [× 10^−3^mm^2^/s]	Dy_proj_ [× 10^−3^mm^2^/s]	Dy_assoc_ [× 10^−3^mm^2^/s]	Dz_proj_ [× 10^−3^mm^2^/s]	Dz_assoc_ [× 10^−3^mm^2^/s]	ALPS index
NC group	Reader 1	0.493 ± 0.131	0.505 ± 0.211	0.504 ± 0.134	0.551 ± 0.181	0.611 ± 0.148	0.403 ± 0.161	1.582 ± 0.221
	Reader 2	0.456 ± 0.159	0.427 ± 0.193	0.410 ± 0.130	0.623 ± 0.231	0.687 ± 0.197	0.517 ± 0.161	1.536 ± 0.204
	ICC	0.931	0.825	0.932	0.811	0.912	0.866	0.884
	95% CI	0.871–0.990	0.738–0.912	0.892–0.972	0.785–0.836	0.889–0.935	0.805–0.967	0.796–0.972
iNPH group	Reader 1	0.533 ± 0.134	0.431 ± 0.125	0.589 ± 0.159	0.511 ± 0.137	0.574 ± 0.181	0.388 ± 0.130	0.945 ± 0.261
	Reader 2	0.661 ± 0.159	0.499 ± 0.211	0.493 ± 0.125	0.685 ± 0.166	0.624 ± 0.140	0.474 ± 0.143	1.203 ± 0.215
	ICC	0.862	0.779	0.836	0.780	0.878	0.853	0.774
	95% CI	0.811–0.913	0.712–0.886	0.797–0.874	0.713–0.847	0.834–0.922	0.784–0.922	0.736–0.812

Values are shown as mean ± SD

*NC* normal control, *iNPH* idiopathic normal pressure hydrocephalus, *ICC* interclass correlation coefficient, *CI* confidence interval, *Dx*_*proj*_ diffusivity along the *x*-axis in projection fibre area, *Dx*_*assoc*_ diffusivity along the *x*-axis in association fibre area, *Dy*_*proj*_ diffusivity along the *y*-axis in projection fibre area, *Dy*_*assoc*_ diffusivity along the *y*-axis in association fibre area, *Dz*_*proj*_ diffusivity along the *z*-axis in projection fibre area, *Dz*_*assoc*_ diffusivity along the *z*-axis in association fibre area

**Table 3:** Comparison of the diffusivities and ALPS index

Diffusivity	NC (*n* = 40)	iNPH (*n* = 41)	*p*-values
Dx_proj_ [× 10^−3^ mm^2^/s]	0.475 ± 0.058	0.597 ± 0.236	0.003*
Dx_assoc_ [× 10^−3^ mm^2^/s]	0.466 ± 0.065	0.465 ± 0.204	0.484
Dy_proj_ [× 10^−3^ mm^2^/s]	0.457 ± 0.118	0.541 ± 0.239	0.152
Dy_assoc_ [× 10^−3^ mm^2^/s]	0.587 ± 0.054	0.598 ± 0.191	0.095
Dz_proj_ [× 10^−3^ mm^2^/s]	0.580 ± 0.073	0.700 ± 0.242	< 0.001*
Dz_assoc_ [× 10^−3^ mm^2^/s]	0.435 ± 0.111	0.479 ± 0.216	0.491
ALPS index	1.559 ± 0.161	1.074 ± 0.225	< 0.001*

Values are shown as mean ± SD

*NC* normal control, *iNPH* idiopathic normal pressure hydrocephalus, *Dx*_*proj*_ diffusivity along the *x*-axis in projection fibre area, *Dx*_*assoc*_ diffusivity along the *x*-axis in association fibre area, *Dy*_*proj*_ diffusivity along the *y*-axis in projection fibre area, *Dy*_*assoc*_ diffusivity along the *y*-axis in association fibre area, *Dz*_*proj*_ diffusivity along the *z*-axis in projection fibre area, *Dz*_*assoc*_ diffusivity along the *z*-axis in association fibre area

* *p*-values less than 0.05 indicate statistical significance

### Associations of diffusivity with WMH and ventricular volume in iNPH

**Fig. 3:**
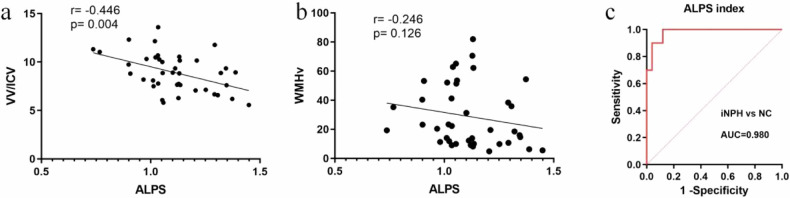
Correlation analysis of ALPS index with ventricular volume and WMH in iNPH patients. **a** Scatterplots describing the associations between the ALPS index and normalised VV in 41 patients with iNPH. **b** Scatterplots describing the associations between the ALPS index and WMH volume in patients with iNPH. **c** ROC curve analysis between the normal control and iNPH groups. iNPH, idiopathic normal pressure hydrocephalus; VV, ventricular volume; ICV, intracranial volume; WMHv, WMH volume; ROC, receiver operating characteristic

As shown in Table 4, the ALPS index was significantly correlated with VV (*r* = −0.451, *p* = 0.024) and normalised VV (*r* = −0.446, *p* = 0.004), but not with total WMH volume (*r* = −0.246, *p* = 0.126) or pWMH (*r* = −0.109, *p* = 0.597). Dx_proj_ was significantly correlated with normalised VV (*r* = 0.391, *p* = 0.049), whereas Dz_proj_ was significantly correlated with WMH volume (*r* = 0.546, *p* = 0.005) and normalised WMH volume (*r* = 0.622, *p* = 0.001). Further regression analyses indicated that after adjusting for important confounders, a reduced ALPS index was independently associated with an increased VV (β = −7.158, *p* = 0.016) (Table 5). However, no association was found between the ALPS index and normalised WMH volume. The ROC curve analysis revealed that the ALPS index discriminates well between iNPH and NCs when applying an optimal cut-off point of 1.322 (sensitivity, 100.0%; specificity, 87.5%; AUC = 0.980) (Fig. 3).

**Table 4:** Correlations between demographics, MRI characteristics, diffusivities and ALPS index in iNPH

	ALPS index	Dx_proj_	Dx_assoc_	Dy_proj_	Dy_assoc_	Dz_proj_	Dz_assoc_
Age	0.062 (0.764)	0.304 (0.132)	0.298 (0.139)	0.118 (0.567)	0.066 (0.749)	0.261 (0.198)	0.217 (0.288)
Sex	0.312 (0.121)	−0.140 (0.495)	−0.006 (0.724)	−0.306 (0.128)	−0.228 (0.261)	−0.234 (0.251)	−0.254 (0.210)
VV	−0.451 (0.024)*	0.387 (0.052)	−0.160 (0.564)	0.282 (0.162)	−0.260 (0.200)	0.348 (0.082)	0.324 (0.106)
VV/ICV	−0.446 (0.004)*	0.391 (0.049)*	−0.111 (0.590)	0.285 (0.171)	−0.185 (0.365)	0.394 (0.048)	0.333 (0.097)
WMHv	−0.246 (0.126)	0.269 (0.183)	0.032 (0.879)	0.390 (0.050)	0.175 (0.392)	0.546 (0.005)*	0.288 (0.154)
WMHv/ICV	−0.251 (0.216)	0.329 (0.101)	−0.042 (0.838)	0.399 (0.050)	0.214 (0.293)	0.622 (0.001)*	0.267 (0.187)
pWMH grade	−0.109 (0.597)	0.325 (0.106)	0.081 (0.695)	0.219 (0.282)	0.365 (0.067)	0.438 (0.029)*	0.188 (0.359)
dWMH grade	−0.160 (0.435)	0.259 (0.202)	0.037 (0.857)	0.335 (0.095)	0.247 (0.224)	0.408 (0.043)*	0.282 (0.163)

Values are shown as *r* (*p*)

*iNPH* idiopathic normal pressure hydrocephalus, *VV* ventricular volume, *ICV* intracranial volume, *WMHv* WMH volume, *pWMH* periventricular white matter hyperintensities, *dWMH* deep white matter hyperintensities, *Dx*_*proj*_ diffusivity along the *x*-axis in projection fibre area, *Dx*_*assoc*_ diffusivity along the *x*-axis in association fibre area, *Dy*_*proj*_ diffusivity along the *y*-axis in projection fibre area, *Dy*_*assoc*_ diffusivity along the *y*-axis in association fibre area, *Dz*_*proj*_ diffusivity along the *z*-axis in projection fibre area, *Dz*_*assoc*_ diffusivity along the *z*-axis in association fibre area

* *p*-values less than 0.05 indicate statistical significance

**Table 5:** Association of ALPS index with WMH and ventricular volume in iNPH

ln VV/ICV ratio as outcome (multiple regression models)				ln WMHv/ICV ratio as outcome (multiple regression models)			
Predictors	β	95% CI	*p*-value	Predictors	β	95% CI	*p*-value
ALPS index	−7.158	(−12.469, −1.847)	0.016*	ALPS index	−2.796	(−6.899, 2.105)	0.161
Age	0.040	(−0.088, 0.168)	0.544	Age	0.068	(−0.047, 0.196)	0.129
Gender	0.470	(−1.260, 2.200)	0.600	Gender	−0.754	(−2.264, 0.899)	0.204
WMHv	−0.024	(−0.083, 0.010)	0.105	VV/ICV	−0.206	(−0.445, 0.211)	0.124

*iNPH* idiopathic normal pressure hydrocephalus, *VV* ventricular volume, *ICV* intracranial volume, *WMHv* WMH volume, *CI* confidence interval

* *p*-values less than 0.05 indicate statistical significance

## Discussion

This study investigated water diffusion along the perivascular space of parenchymal vessels, i.e., the ALPS index, based on DTI to assess the activity of the glymphatic system in individual cases; compared the ALPS index between patients with iNPH and age- and sex-matched NCs; and subsequently assessed the association of ALPS index with VV and WMH. This study found a significantly reduced ALPS index in patients with iNPH. Furthermore, the decline in ALPS index was independently associated with an increased VV. However, no correlation was identified between the ALPS index and WMH. These findings suggest that a reduced ALPS index may indicate glymphatic dysfunction in patients with iNPH, with ventriculomegaly being a potential associated factor.

The reduced ALPS index in patients with iNPH is consistent with the findings of several previous studies [9–12], suggesting decreased water diffusion along the perivascular space. Glymphatic MRI, the gold standard for assessing glymphatic function, has confirmed glymphatic impairment in iNPH [4]. A recent study showed that the ALPS index is significantly correlated to glymphatic clearance function calculated based on intrathecal enhanced MRI [19]. Thus, the ALPS index can serve as a non-invasive indicator of glymphatic dysfunction in patients with iNPH. However, as an indirect measure of glymphatic function, the ALPS index may not accurately reflect the true glymphatic activity due to disease itself or other confounding factors [20]. Therefore, caution is required when inferring glymphatic impairment in iNPH.

The ALPS index exhibited a negative correlation with VV, which is consistent with two prior studies [9, 12]. However, only a few studies have focused on this relationship. Kikuta et al [11] recently investigated changes in the ALPS index based on DTI after shunt surgery. They found that the mean ALPS index was significantly higher postoperatively than preoperatively in patients with iNPH, especially in the responder group, suggesting a possible recovery of glymphatic function accompanied by decreased ventricular volume after surgery. Similarly, Georgiopoulos et al [12] recently found a strong negative correlation between the Evans index and the ALPS index. Accumulating evidence indicates that the glymphatic system plays a role in CSF absorption and circulation, and abnormal CSF dynamics contribute to ventricular enlargement in iNPH [21]. These observations prompt speculation regarding the potential impact of glymphatic dysfunction on ventriculomegaly. Notably, our study adds new support to this hypothesis. Although the pathogenesis of iNPH remains unclear, it is characterised by secondary restricted arterial pulsation caused by reduced intracranial compliance [21, 22]. Glymphatic dysfunction and ventriculomegaly may share common pathophysiological features. Experimental studies in mice suggested that arterial pulsation drives fluid flow in the perivascular space [23]. Consequently, iNPH may cause water diffusion failure in the perivascular space, resulting in glymphatic dysfunction. Furthermore, limited pulsation and subsequent increases in capillary pulsations may increase ventricular pulse pressure and pulsatile CSF flow in the aqueduct and dilate the ventricles [21].

WMH is another typical imaging marker in patients with iNPH. Since the introduction of the ALPS index, few studies have explored the relationship between the ALPS index and WMH in iNPH. We observed increased WMH volume, pWMH, and dWMH Fazekas scores with decreasing ALPS index; however, this trend was not significant. Several studies have demonstrated that glymphatic function is independently associated with the WMH volume in cerebral small vessel disease (cSVD), indicating that WMH is one imaging marker of cSVD. The cerebral glymphatic function is associated with the clearance of cerebral metabolites, and its dysfunction leads to decreased clearance of inflammatory cells and factors from the brain, resulting in an abnormal accumulation of dissolved and metabolic wastes, which further triggers hypoxic and inflammatory responses in the surrounding tissues and eventually cause brain parenchymal injury. Previous studies have shown that inflammation is involved in WMH development [24, 25]. However, there is no relationship between the ALPS index and WMH in patients with iNPH, which is consistent with previous findings by Georgiopoulos et al [12]. Due to ventricular dilatation and altered CSF pressure gradients in patients with iNPH, increased resistance to glymphatic flow may also cause CSF to follow the path of least resistance, including the retrograde transventricular route, resulting in an increased accumulation of CSF in the deep and periventricular white matter [4], and therefore the measured ALPS index may not reflect the true glymphatic function [20]. Moreover, diffusion microstructure imaging has revealed a significant increase in the free water fraction within the pWMH in a cohort exhibiting iNPH imaging features [26]; the finding supports the hypothesis of increased accumulation of free water, particularly around the periventricular areas, in the brain parenchyma in patients with iNPH [27]. This mechanism could account for the association between increased Dz_proj_ and higher WMH Fazekas scores and volume in this study.

However, the aetiology of dWMH is complex and can be challenging to differentiate from that of lacunar infarcts. The accumulation of dWMH could stem from the deposition of toxic waste products due to glymphatic dysfunction [28]. Additionally, dWMH may also be associated with blood-brain barrier dysfunction [29] or ageing. The coexistence of multiple pathological mechanisms of WMH may explain the absence of a link between the ALPS index and the WMH volume or pWMH and dWMH Fazekas scores. Additionally, according to the DTI-ALPS index measurements, diffusivities were only available in the area outside the lateral ventricle along the plane of the lateral ventricular body. Therefore, the evaluation of perivascular diffusivity in other regions where the perivascular space does not align with the *x*-, *y*-, or *z*-axes is challenging. Despite the ubiquitous presence of the perivascular space throughout the brain, it is currently challenging to assess alterations in the overall ALPS index. Thus, the periventricular area might be the optimal area for assessing glymphatic dysfunction in iNPH [5].

This study has some limitations. First, its cross-sectional nature precludes the determination of causality. Second, the study’s retrospective design, its execution at a single site, and the involvement of a small cohort further limit the generalisability of the findings. Further studies with larger and more diverse cohorts are needed to comprehensively evaluate glymphatic dysfunction and its potential contributing factors, such as ageing.

In conclusion, using the non-invasive MRI-based DTI-ALPS index method, we found that a reduced ALPS index may indicate glymphatic impairment in iNPH. Moreover, a lower ALPS index was associated with decreased glymphatic activity and ventriculomegaly but not with WMH. These findings may reveal some potential factors affecting the occurrence of glymphatic dysfunction in iNPH. Future studies should investigate longitudinal changes in ALPS index after shunt surgery and a potential correlation between the ALPS index and disease severity of iNPH patients.

## Abbreviations

DTI-ALPS: Diffusion tensor image analysis along the perivascular space
dWMH: Deep white matter hyperintensity
FA: Fractional anisotropy
FLAIR: Fluid-attenuated inversion recovery
ICV: Intracranial volume
iNPH: Idiopathic normal pressure hydrocephalus
MPRAGE: Magnetisation-prepared rapid acquisition gradient-echo
NCs: Normal controls
pWMH: Periventricular white matter hyperintensity
ROC: Receiver operating characteristic
ROIs: Regions of interest
SWI: Susceptibility-weighted imaging
VV: Ventricular volume
WMH: White matter hyperintensity
